# Antibiotic-induced gut microbiota disruption promotes vascular calcification by reducing short-chain fatty acid acetate

**DOI:** 10.1186/s10020-024-00900-0

**Published:** 2024-08-24

**Authors:** Shi-Yu Zeng, Yi-Fu Liu, Zhao-Lin Zeng, Zhi-Bo Zhao, Xi-Lin Yan, Jie Zheng, Wen-Hang Chen, Zhen-Xing Wang, Hui Xie, Jiang-Hua Liu

**Affiliations:** 1 Department of Metabolism and Endocrinology, The First Affiliated Hospital, Hengyang Medical School, University of South China, Hengyang, 421001 Hunan China; 2 Department of Orthopedics, Movement System Injury and Repair Research Center, Xiangya Hospital, Central South University, Changsha, 410008 Hunan China; 3 Hunan Key Laboratory of Angmedicine, Changsha, 410008 Hunan China; 4 National Clinical Research Center for Geriatric Disorders, Xiangya Hospital, Central South University, Changsha, 410008 Hunan China; 5 The Second Affiliated Hospital, Department of Urology, Hengyang Medical School, University of South China, Hengyang, Hunan China; 6 Department of Urology, The First Affiliated Hospital of Nanchang University, Nanchang, 330000 Jiangxi China; 7 Hunan Diabetes Clinical Medical Research Center, Hengyang, 421001 Hunan China; 8 Department of Nephrology, Xiangya Hospital, Central South University, Changsha, 410008 Hunan China

**Keywords:** Antibiotic, Vancomycin, Gut microbiota, Vascular calcification, Short-chain fatty acid, Acetate

## Abstract

**Background:**

Vascular calcification is a common vascular lesion associated with high morbidity and mortality from cardiovascular events. Antibiotics can disrupt the gut microbiota (GM) and have been shown to exacerbate or attenuate several human diseases. However, whether antibiotic-induced GM disruption affects vascular calcification remains unclear.

**Methods:**

Antibiotic cocktail (ABX) treatment was utilized to test the potential effects of antibiotics on vascular calcification. The effects of antibiotics on GM and serum short-chain fatty acids (SCFAs) in vascular calcification mice were analyzed using 16 S rRNA gene sequencing and targeted metabolomics, respectively. Further, the effects of acetate, propionate and butyrate on vascular calcification were evaluated. Finally, the potential mechanism by which acetate inhibits osteogenic transformation of VSMCs was explored by proteomics.

**Results:**

ABX and vancomycin exacerbated vascular calcification. 16 S rRNA gene sequencing and targeted metabolomics analyses showed that ABX and vancomycin treatments resulted in decreased abundance of *Bacteroidetes* in the fecal microbiota of the mice and decreased serum levels of SCFAs. In addition, supplementation with acetate was found to reduce calcium salt deposition in the aorta of mice and inhibit osteogenic transformation in VSMCs. Finally, using proteomics, we found that the inhibition of osteogenic transformation of VSMCs by acetate may be related to glutathione metabolism and ubiquitin-mediated proteolysis. After adding the glutathione inhibitor Buthionine sulfoximine (BSO) and the ubiquitination inhibitor MG132, we found that the inhibitory effect of acetate on VSMC osteogenic differentiation was weakened by the intervention of BSO, but MG132 had no effect.

**Conclusion:**

ABX exacerbates vascular calcification, possibly by depleting the abundance of *Bacteroidetes* and SCFAs in the intestine. Supplementation with acetate has the potential to alleviate vascular calcification, which may be an important target for future treatment of vascular calcification.

**Supplementary Information:**

The online version contains supplementary material available at 10.1186/s10020-024-00900-0.

## Introduction

Vascular calcification is an extremely common phenomenon in the human body, which is caused by calcium phosphate deposition as a result of multiple complex pathophysiologic processes (Sutton et al. 2023). Previously, it was believed that the progression of vascular calcification is mainly dependent on age, and increasing evidence suggests that it is also strongly associated with atherosclerosis, hypercholesterolemia, diabetes mellitus, hypertension, and chronic kidney disease (Pan et al. 2023). One study found that among 4,025 general subjects with a mean age of 59.4 years, 2,538 participants (63.1%) had thoracic aortic calcification, and 74% of this group had concomitant coronary artery calcification (Kälsch et al. 2013). Epidemiologic investigations have shown that the more severe the abdominal aortic and coronary artery calcification, the higher the morbidity and mortality from coronary events (Roe et al. 2010; Chen et al. 2018). This evidence suggests that vascular calcification has become a serious threat to human health, especially in elderly patients with concomitant risk factors. Unfortunately, however, there is still a lack of effective therapeutic strategies to reverse vascular calcification (Pan et al. 2023). Therefore, efforts to explore the underlying pathogenesis to slow the progression of vascular calcification are essential.

The gut microbiota (GM) has been identified as an integral component in maintaining host metabolic and immune homeostasis (Fan and Pedersen 2021; Lu et al. 2022). The composition and structure of GM is influenced by host age, disease, living environment, dietary habits and drug use, and alterations in GM due to these factors can also affect the immune and metabolic microenvironments of organs in the body, including: the gut, brain, liver, bone and heart. In previous studies, the relationship between GM and atherosclerosis has been widely explored and some promising therapeutic targets been identified, such as the metabolites of gut microbes Trimethylamine-N-oxide (TMAO) and Indole-3-Propionic Acid (Ma et al. 2022; Xue et al. 2022). Similarly, TMAO has been found to promote vascular calcification through activation of NLRP3 inflammatory vesicles and NF-κB signaling (Zhang et al. 2020), whereas short-chain fatty acid (SCFA) propionate derived from GM may alleviate vascular calcification by remodeling the GM (Yan et al. 2022). Besides, recent evidence suggests that alpha diversity in the fecal microbiota is significantly lower and the microbiota dysbiosis index is elevated in patients with high aortic arch calcification compared with those with low aortic arch calcification (Liu et al. 2023). Based on these results, we hypothesized that the homeostasis of the GM may be closely related to the progression of vascular calcification.

Antibiotics are undoubtedly the most used drugs in clinical practice, especially given the persistent antibiotic abuse scenario (Akram et al. 2023). In addition to treating bacterial infections, they are unavoidably accompanied by side effects such as GM disruption (Duan et al. 2022). Notably, antibiotic-induced GM disorders have been shown to be associated with the progression of several diseases and the response to cancer treatment. For instance, antibiotic-induced GM disruption exacerbates cholestatic liver injury (Schneider et al. 2021), inhibits liver regeneration (Yin et al. 2023), promotes cognitive deficits in diabetic mice (Zheng et al. 2021) and confers cisplatin resistance in epithelial ovarian cancer (Chambers et al. 2022). However, evidence regarding the effect of antibiotic-induced GM depletion or disruption on vascular calcification remains elusive to date.

Here, we explored whether antibiotic treatment could exacerbate vascular calcification in a mouse model of vitamin D (VD) - induced vascular calcification, and analyzed the effects of antibiotics on the fecal microbiota and related metabolites in mice using 16 S ribosomal RNA (rRNA) gene sequencing and targeted metabolomics. We then explored the effects of the candidate metabolites on vascular calcification in vivo and analyzed their effects on the osteogenic transformation in vascular smooth muscle cells (VSMCs) in vitro. Finally, we explored the potential mechanisms by which the candidate metabolites inhibit osteogenesis in VSMCs using proteomics.

## Materials and methods

### Animals and treatment

All experimental animals were conducted in accordance with the Chineses animal protection law and the National Institutes of Health (NIH) Guide for the Care and Use of Laboratory Animals. The experimental animal protocols were reviewed and approved by the Ethics Committee of South China University (protocol number: USC2023XS038). 8-week-old C57BL/6 male mice were used in this study. Antibiotic cocktail (ABX) (including vancomycin (0.5 g/L), metronidazole (1 g/L), ampicillin (1 g/L), neomycin (1 g/L) (Macklin, Shanghai, China) and SCFAs (sodium acetate, NaAce (200mM); sodium propionate, NaPro (200mM), sodium butyrate, NaBut (200mM) (Solarbio, Beijing, China) were added to drinking water. After 4 weeks of intervention, Feces from antibiotic-treated or untreated mice were collected for 16 S rRNA and SCFAs analysis. Mice were intraperitoneally injected with VD (750 U/kg/d) for 4 days to establish a vascular calcification model (Li et al. 2023; Wang et al. 2022). Finally, mice were euthanized by a single intraperitoneal injection of sodium pentobarbital (200 mg/kg), and blood samples and aortic samples were collected for further testing.

### Fecal microbiota transplantation

The feces were transplanted as described in previous studies (Chen et al. 2022). Briefly, the feces of mice with or without antibiotic treatment at 4 weeks were collected. Feces were resuspended in PBS and centrifuged at 500 g for 1 min to collect the supernatant. The microbiota-depleted recipients were administered supernatant orally three times a week. All mice received intraperitoneal injections of VD for 4 consecutive days and were sacrificed ten days later.

### Determination of calcium content

The aortas and VSMCs were decalcified by 5% HCI for 48 h at 4℃, and then the HCI supernatant was collected for calcium content detection with a calcium Ion Assay Kit (Beyotime, Shanghai, China). For normalizing calcium content, aortic protein concentration was assessed using BCA method (Elabscience, Wuhan, China), and calcium content was expressed as µmol/ug vessel protein.

**Alizarin red staining**.

For whole-mount aorta staining, the dissected aortas were fixed in 95% ethanol for 24 h at 37℃. Staining solution was prepared by mixing 0.5 mL 0.1% alizarin red with 50 mL 1% potassium hydroxide. Aortas were stained at room temperature overnight. The aortas were washed twice with 2% potassium hydroxide before imaging. For section aorta staining, 5 μm-thick aortic sections were hydrated and then stained with alizarin red.

### Cell culture

VSMCs were grown in complete medium containing DMEM/F12 (Procell, Wuhan, China), 10% fetal bovine serum (FBS) and 1% penicillin–streptomycin. VSMCs were used for subsequent experiments at passages 3 to 8. VSMCs were seeded into 24-well or 48-well plates at an initial density of 5*10^4 or 2*10^4 cells per well, respectively. When grew to 70-80% confluence, the DMEM/F12 complete medium was replaced with the osteogenic medium (OS) supplemented with NaAce (10mM), Buthionine sulfoximine (BSO) (25 μm) (S9728) and MG132 (10 μm) (S2619) for 5 days.

### Alkaline phosphatase (ALP) staining

VSMCs were removed from culture medium and washed three times with PBS. VSMCs were fixed with 4% paraformaldehyde for 20 min at room temperature and then washed three times with PBS. Finally, VSMCs were incubated with ALP staining solution (Beyotime, Shanghai, China) at room temperature in the dark for 2–24 h.

### Immunofluorescence staining

VSMCs were plated onto coverslips in 24-well plates and allowed to incubate overnight. Following the designated treatment, the cells were fixed with 4% PFA for 20 min and permeabilized using 0.3% Triton X-100 for 30 min at room temperature. The cells were then blocked with 5% bovine serum albumin for 1 h and incubated with a primary antibody targeting RUNX2 (diluted 1:1600; Cell Signaling Technology) and BMP2 ((diluted 1:200; Abcam)overnight at 4 °C. Afterward, the cells were incubated with a secondary antibody (diluted 1:300; donkey anti-rabbit Alexa Fluor 488) for 1 h at room temperature in the dark. The nuclei were stained with DAPI for 5 min and subsequently visualized using fluorescent microscope.

### qRT-PCR analysis

RNA was extracted using Ultrapure RNA Kit (Cowin Bio, Taizhou, China) according to the manufacturer’s instruction. The extracted RNA was reverse transcribed using cDNA Synthesis SuperMix (Novoprotein, Suzhou, China).). Then, qRT-PCR was performed using SYBR Green qPCR Master Mix (Bimake, Shanghai, China). The sequences of RNAs were as follows: Runx2, 5’- TGGTTACTGTCATGGCGGGTA − 3’ (forward) and 5’- TCTCAGATCGTTGAACCTTGCTA − 3’ (reverse), Alpl, 5’- ACTGGTACTCAGACAACGAGAT − 3’ (forward) and 5’- ACGTCAATGTCCCTGATGTTATG − 3’ (reverse), αSMA, 5’- TGCCAACAACGTCATGTCG − 3’ (forward) and 5’- CAGCGCGGTGATCTCTTTCT − 3’ (reverse).

### 16s rRNA gene sequencing

After 4 weeks of ABX and various antibiotic interventions, fresh stool samples were collected for each individual mouse and immediately snap-frozen in liquid nitrogen before storage at -80℃ until DNA extraction. The stool sample was sent to Genesky Biotechnologies Inc. (Shanghai, China) for 16s rRNA Gene Sequencing. Brifely, afte extraction of stool DNA, the V3–V4 region of the 16 S rRNA gene was amplified by PCR, the uniqueness and specificity of the amplified products were detected by agarose gel electrophoresis. Then, the specific tag sequences were added to each sample, and the specific tag sequences were introduced to the end of the library by PCR. The amplified products were purified by nucleic acid purification magnetic beads, and an original library of samples was obtained. According to the preliminary quantitative results of agarose gel electrophoresis, the concentration of the sample libraries with their respective index labels was appropriately diluted, and the libraries were accurately quantified using Qubit. The samples were mixed according to the sequencing flux requirements of different samples. The size of the inserted fragment of the sequencing library was detected by Agilent 2100 BIoanalyzer to confirm that there was no specific amplification between 120 and 200 bp, and the concentration of the sequencing library was accurately quantified. Finally, the library was sequenced using NovaSeq 6000 platform and SP-Xp (PE250) double-ended sequencing strategy.

### Western blotting analysis

Equal amounts of protein samples were separated by SDS/PAGE gels and transferred to polyvinylidene difluoride (PVDF) membranes. After blocking with 5% BSA for 1 h, the PVDF membrane was incubated with the specific primary antibody overnight at 4 °C. The membrane was washed three times with PBST and then incubated with HRP-linked goat anti-rabbit secondary antibody for 1 h at room temperature. The signals were detected by ChemiDocTM Touch Imaging System (BIO-RAD) with an ECL kit.

### SCFAs measurement

An equal amount of 50% aqueous acetonitrile was added to mouse serum (90 µL), and vortexing, ultrasonic extraction, and centrifugation were performed sequentially, followed by transferring 80 µL of the supernatant to an injection vial. Then the samples and standards were derivatized, and the content of SCFAs in mouse serum was determined using a liquid chromatography mass spectrometer (LCMS) system with the aid of high-performance liquid chromatography (HPLC, Nexera UHPLC LC-30 A, Japan) and high-sensitivity mass spectrometer (AB Sciex Qtrap 5500, USA).

### Proteomic analysis

Label-free proteomic analysis of three biological replicates from Vehicle (NC1, NC2 and NC3), Vehicle + OS (OS1, OS2 and OS3) and NaAce + OS groups (Ace_OS1, Ace_OS2, Ace_OS3) was carried out by Giga Genomics (Shanghai, China). Briefly, total proteins of VSMCs were extracted, digested with trypsin (50:1) overnight, and tryptic peptides were dissolved in 0.1% formic acid and 2% acetonitrile, and then chromatographed and separated on an EASY-nLC 1000 ultra-performance liquid chromatograph at a flow rate of 500 nl/min. The obtained peptides were subjected to nanospray ionization and then analyzed by tandem mass spectrometry (MS/MS) in a Q Exactive Plus (Thermo Fisher Scientific).

### Statistical analysis

The data in this study were expressed as mean ± standard deviation (SD), and statistical analysis was performed using GraphPad Prism 8.0 software. A two-tailed Student’s *t*-test was used to compare the two groups of data that conformed to a normal distribution; otherwise, the Mann-Whitney U test was used. One -way analysis of variance (ANOVA) test, or Kruskal Wallis test was used for comparisons among multiple groups and *P* < 0.05 was set as statistically significant.

## Results

### Antibiotic cocktail treatment exacerbates VD-induced vascular calcification by modulation of GM

**Fig. 1:**
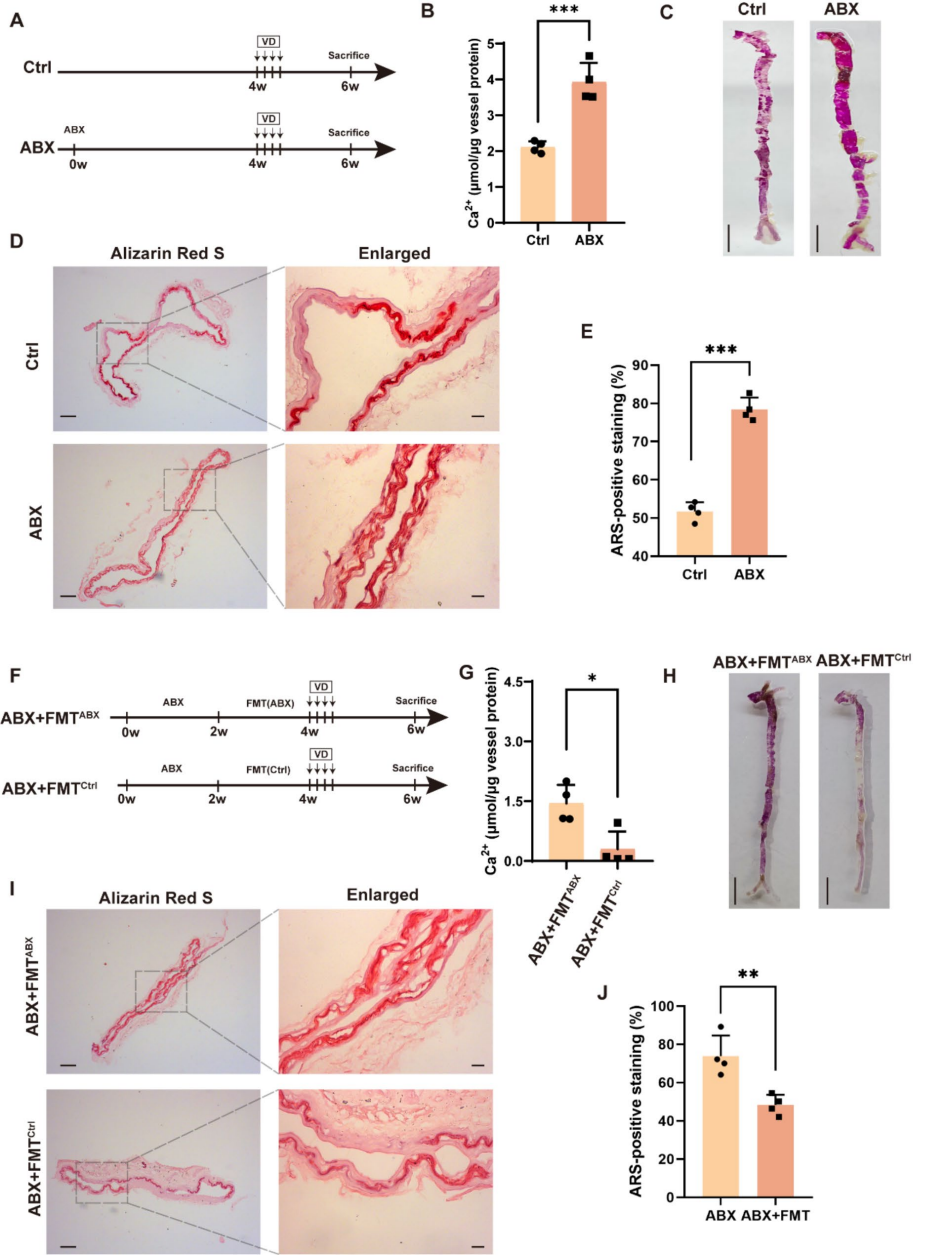
Antibiotic cocktail treatment exacerbates VD-induced vascular calcification in mice by modulation of GM. (**A**) Flow diagram of the antibiotic cocktail (ABX) intervention experiment. (**B**) Quantitative evaluation of aortic calcium content in mice. *n* = 4 per group. (**C**) Macroscopic observation of arterial vascular calcification. Scale bar = 0.5 cm. (**D**) Calcification of ascending aortic vessels based on alizarin red-stained tissue sections. Scale bar = 100 μm (right) and 20 μm (left). (**E**) Relative quantification of positive ARS area in ascending aortic vascular sections. *n* = 4 per group. (**F**) A flow diagram of the fecal microbiota transplantation (FMT) experiment. (**G**) Quantitative evaluation of aortic calcium content in mice. *n* = 4 per group. (**H**) Macroscopic observation of arterial vascular calcification. Scale bar = 0.5 cm. (**I**) Calcification of ascending aortic vessels based on alizarin red-stained tissue sections. Scale bar = 100 μm (right) and 20 μm (left). (**J**) Relative quantification of positive ARS area in ascending aortic vascular sections. *n* = 4 per group. Data are presented as mean ± SD. *P < 0.05, **P < 0.01, and ***P < 0.001

To determine whether antibiotic-induced GM disruption affects vascular calcification, we treated mice with an antibiotic cocktail (ABX) regimen (including vancomycin, metronidazole, ampicillin, neomycin) for 4 weeks prior to VD-induced vascular calcification (Fig. 1A). It was found that mice treated with ABX had significantly higher aortic calcium content and significantly increased calcium salt deposition compared to control mice (Fig. 1B and C). Similarly, alizarin red staining of ascending aortic sections showed significantly enhanced deposition of calcium salts in the vessels of mice treated with ABX (Fig. 1D and E). These results suggest that ABX exacerbates VD-induced vascular calcification in mice.

To explore whether ABX-induced exacerbation of VD is related to GM, we performed fecal microbial transplantation (FMT) on mice in the ABX + FMT^Ctrl^ group with feces from mice in the control group prior to induction of the vascular calcification model, whereas mice in the ABX + FMT^ABX^ group were treated with fecal bacteria from mice in the ABX group (Fig. 1F). As we expected, mice in the ABX + FMT^Ctrl^ group had significantly lower calcium levels and significantly less calcium salt deposition in the aorta compared with the ABX + FMT^ABX^ group (Fig. 1G and H). Moreover, alizarin red staining showed that alleviation of ABX-induced GM disruption with relatively healthy fecal bacteria attenuated calcium salt deposition in the aorta (Fig. 1I and J). Together, ABX may exacerbate VD-induced vascular calcification by disrupting GM.

### Vancomycin treatment exacerbates VD-induced vascular calcification

**Fig. 2:**
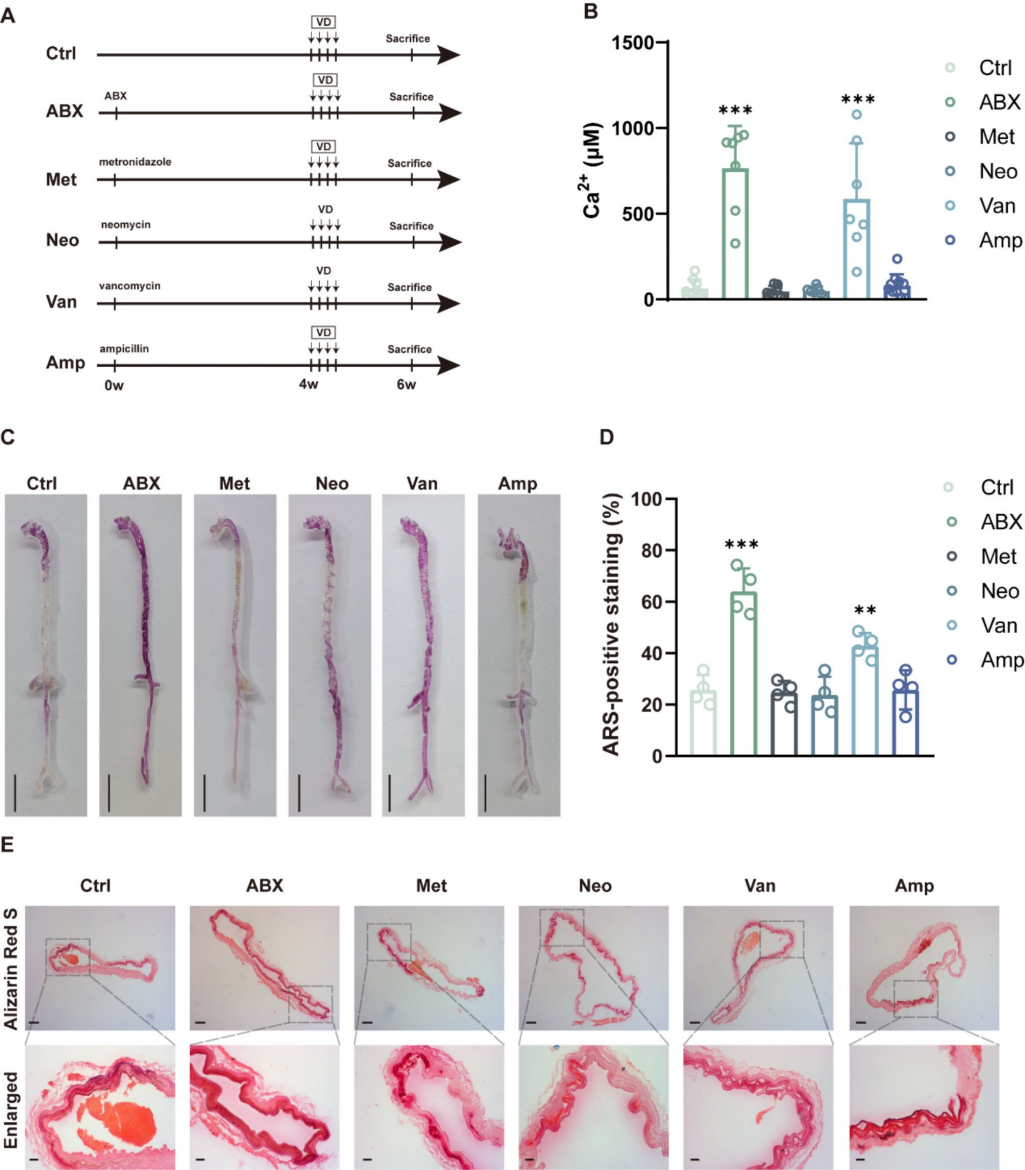
Vancomycin treatment exacerbates VD-induced vascular calcification in mice. (**A**) Flow diagram of the single antibiotic intervention experiment. (**B**) Quantitative evaluation of aortic calcium content in mice. *n* = 7–10 per group. (**C**) Macroscopic observation of arterial vascular calcification. Scale bar = 0.5 cm. (**D**) Relative quantification of positive ARS area in ascending aortic vascular sections. *n* = 4 per group. (**E**) Calcification of ascending aortic vessels based on alizarin red-stained tissue sections. Scale bar = 100 μm (top) and 20 μm (bottom). Data are presented as mean ± SD. *P < 0.05, **P < 0.01, and ***P < 0.001

To further analyze which antibiotic causes increased vascular calcification, we treated mice with vancomycin, metronidazole, ampicillin, and neomycin for 4 weeks before induction of the vascular calcification model (Fig. 2A). As shown in Fig. 2B and C, calcium content and calcium salt deposition were significantly increased in the aorta of mice in the ABX and vancomycin groups, whereas no significant changes were observed in mice treated with metronidazole, ampicillin, and neomycin. Consistent with this, results of alizarin red staining showed increased calcium salt deposition in the ABX- and vancomycin-treated mice but not in the metronidazole, ampicillin, and neomycin group (Fig. 2D and E). These results suggest that vancomycin replicates, at least in part, the pro-vascular calcification effects of ABX, and that the GM disruption it causes may be a key factor in exacerbating vascular calcification.

### Antibiotic treatment alters the structure and composition of GM and reduces the production of GM metabolite SCFAs

**Fig. 3:**
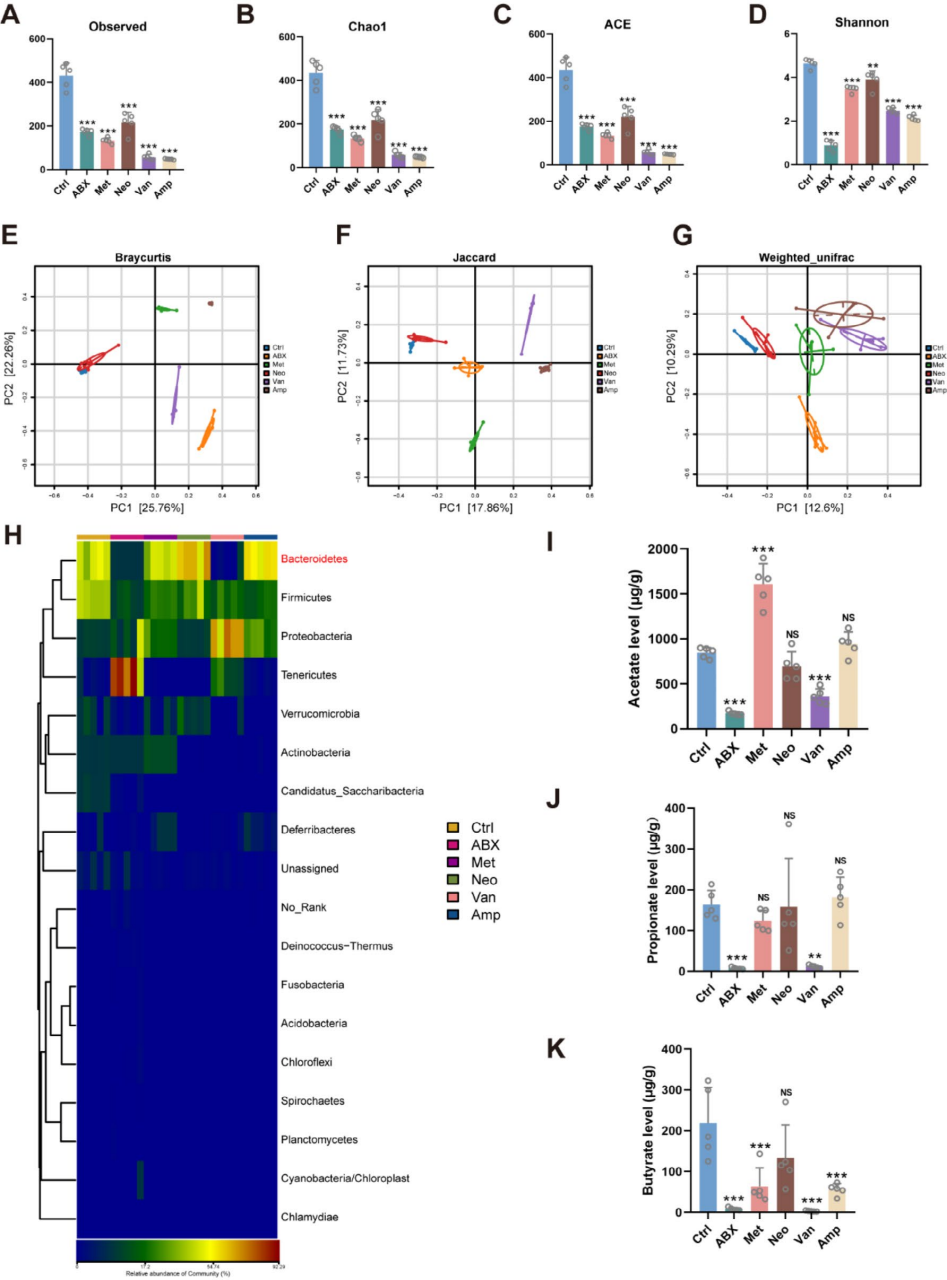
Antibiotic treatment alters the structure and composition of GM and reduces the production of GM metabolite SCFAs. (**A** to **D**) Observed number of OTUs and estimated OTU richness (Chao1, ACE, and Shannon) in fecal microbiota of mice. *n* = 5 per group. (**E** to **G**) Principal coordinate analysis (PCoA) plot based on Braycurtis distances, Jaccard distances and Weighted_unifrac distances. *n* = 5 per group. (**H**) Relative abundance of the identified fecal microbiota at the phylum level as detected by 16 S rRNA gene sequencing. *n* = 5 per group. (**I** to **J**) short-chain fatty acids in mouse serum. *n* = 5 per group. Data are presented as mean ± SD. *P < 0.05, **P < 0.01, and ***P < 0.001

Considering that antibiotic-induced exacerbation of vascular calcification in mice is associated with GM, we analyzed fecal microbiota alterations in antibiotic-treated vascular calcification mice using 16 S rRNA sequencing. First, we assessed the alpha diversity of the fecal microbiota of vascular calcification mice. The observed number of operational taxonomic units (OTUs) and estimated OTU abundance (Chao1, ACE, and shannon) were significantly decreased in the fecal microbiota of the ABX, metronidazole, neomycin, vancomycin, and ampicillin groups compared to the control group, suggesting that antibiotic treatments greatly reduced the species diversity of the fecal microbiota (Fig. 3A to D). Besides, based on principal coordinate analysis (PCoA) we assessed β-diversity in the fecal microbiota, and the results showed that ABX and vancomycin treatments had the greatest effect on on β-diversity, while neomycin had the weakest effect. (Fig. 3E to G). Further, we assessed changes in the composition of the fecal microbiota in each group of mice. At the phylum (Fig. 3H), Class (Fig. S1A), and order levels (Fig. S1B), the abundance of *Bacteroidetes* was significantly decreased in both the ABX and vancomycin groups, whereas there were no significant differences in the other treatment groups. Apparently, this alteration is consistent with increased aortic calcification in mice.

Since *Bacteroidetes* is one of the main microorganisms producing SCFAs (Ma et al. 2020; Hu et al. 2020), we then examined the effect of antibiotics on SCFAs (acetate, propionate and butyrate) in the serum of vascular calcification mice using targeted metabolomics. As expected, ABX and vancomycin treatments significantly reduced the levels of acetate, propionate and butyrate in the serum of mice (Fig. 3I to K). In the metronidazole-treated group, the content of acetate was elevated, that of butyrate was reduced, and that of propionate was not significantly changed (Fig. 3I to K). In addition, neomycin had no significant effect on the production of SCFAs in vascular calcification mice, while ampicillin merely reduced the concentration of butyrate (Fig. 3I to K). Based on this evidence, we hypothesize that the exacerbating effects of ABX and vancomycin on vascular calcification in mice may be related to the fact that the production of SCFAs was reduced.

### NaAce supplementation alleviates VD-induced vascular calcification

**Fig. 4:**
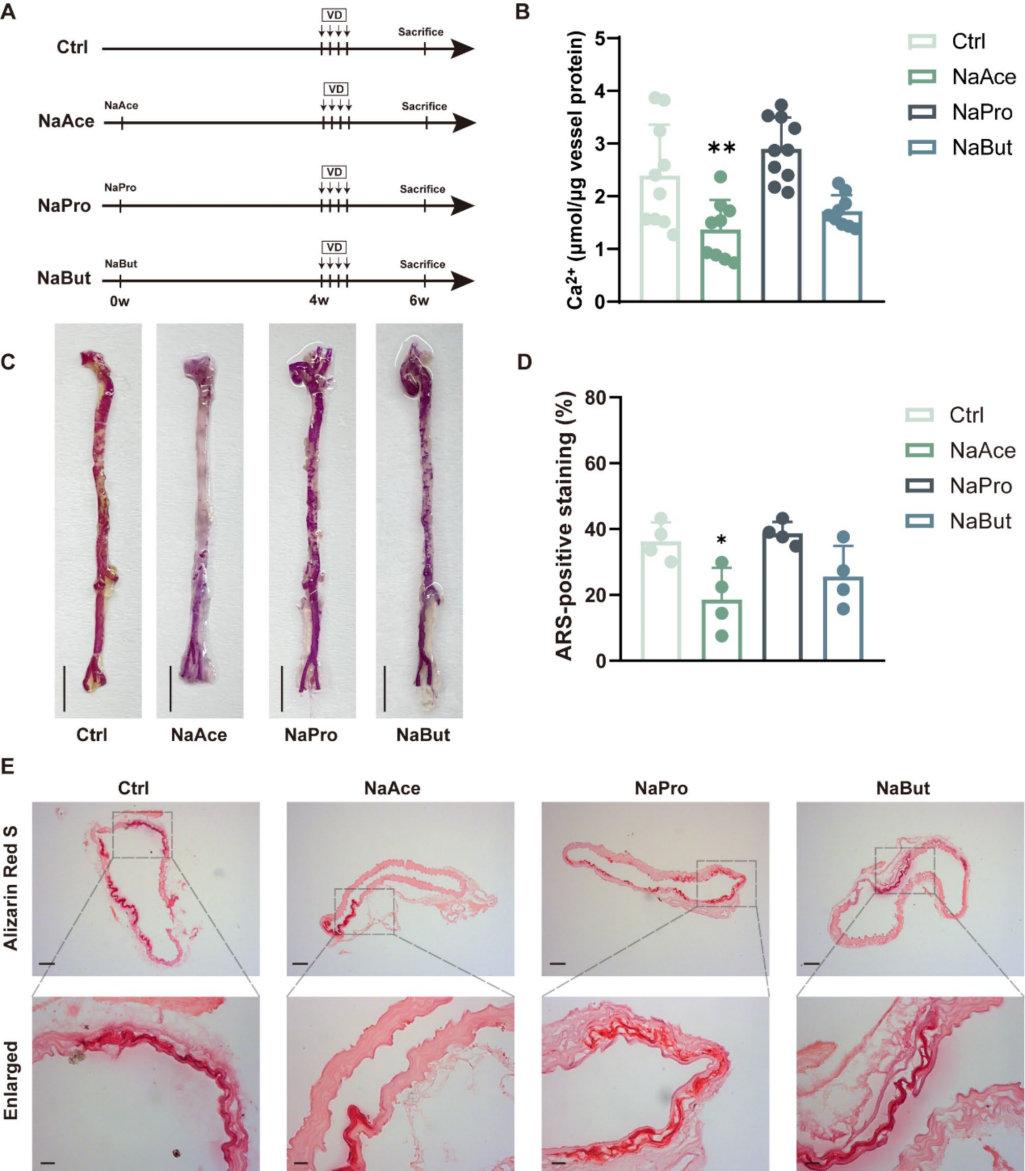
NaAce supplementation alleviates VD-induced vascular calcification in mice. (**A**) Flow diagram of the short-chain fatty acids intervention experiment. (**B**) Quantitative evaluation of aortic calcium content in mice. *n* = 9–10 per group. (**C**) Macroscopic observation of arterial vascular calcification. Scale bar = 0.5 cm. (**D**) Relative quantification of positive ARS area in ascending aortic vascular sections. *n* = 4 per group. (**E**) Calcification of ascending aortic vessels based on alizarin red-stained tissue sections. Scale bar = 100 μm (top) and 20 μm (bottom). Data are presented as mean ± SD. *P < 0.05, **P < 0.01, and ***P < 0.001

To test whether SCFAs could alleviate VD-induced vascular calcification, we treated them with drinking water containing NaAce, NaPro, and NaBut, respectively, for 4 weeks before induction of the vascular calcification model (Fig. 4A). The results showed that NaAce treatment, but not NaPro and NaBut, reduced calcium levels and calcium salt deposition in the aorta of mice (Fig. 4B and C). In addition, alizarin red staining of ascending aortic sections showed a significant reduction in calcium salt deposition in the vessels of mice in the NaAce group, a slight reduction in the NaBut group, and no significant change in the NaPro group (Fig. 4D and E). These results suggest that gut microbe-derived acetate is a protective factor for VD-induced vascular calcification.

### NaAce inhibits osteoblast differentiation of VSMCs in vitro

**Fig. 5:**
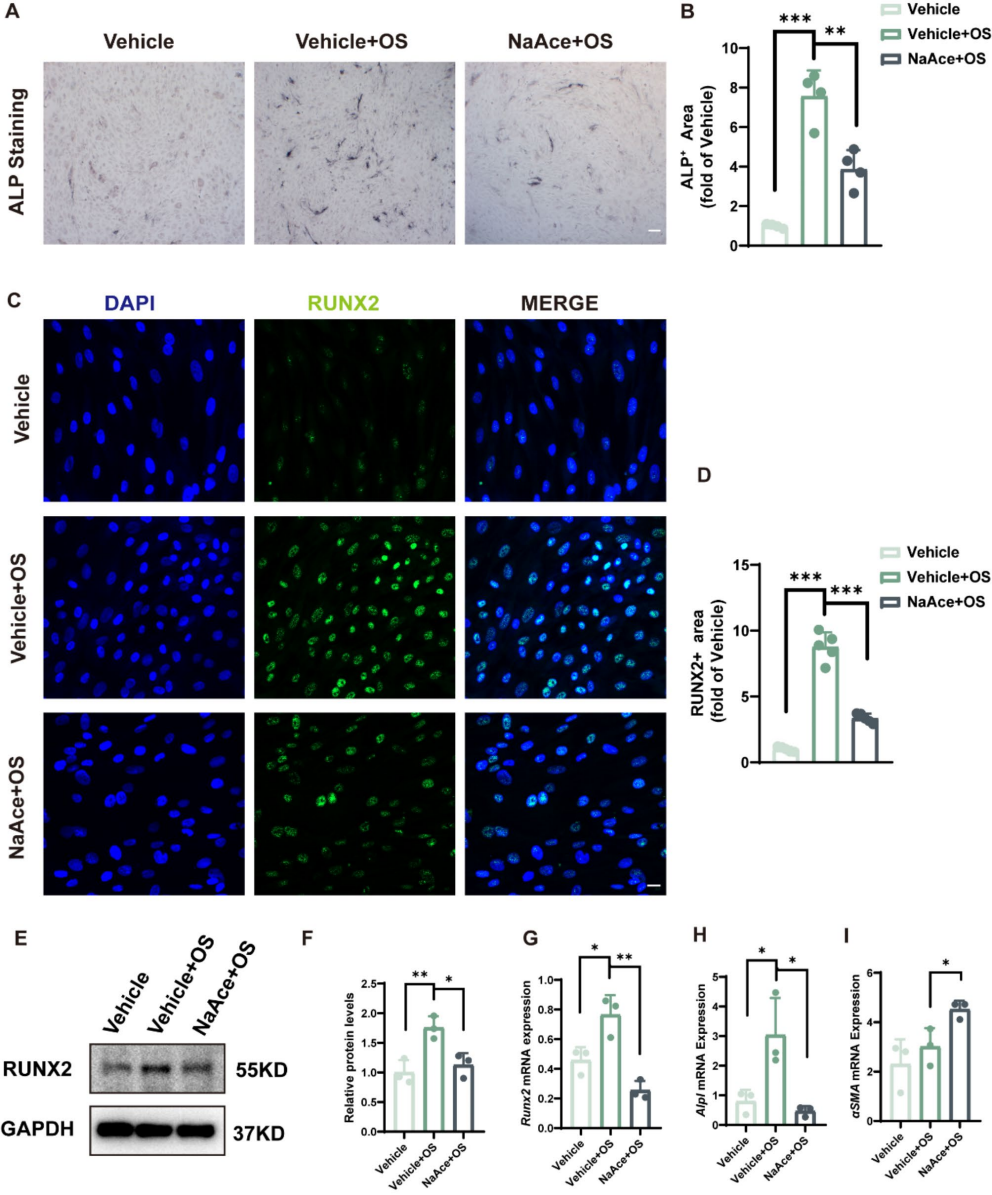
NaAce inhibits osteoblast differentiation of VSMCs in vitro. (**A**) Alkaline phosphatase (ALP) staining images of VSMCs. Scale bar = 200 μm. (**B**) Relative quantification of ALP activity. *n* = 4 per group. (**C**) Immunofluorescence images of Runx2 protein in VSMCs. Scale bar = 20 μm. (**D**) Quantification of relative expression of Runx2 protein in VSMCs. *n* = 5 per group. (**E** and **F**) Protein expression of Runx2 determined using western blotting. *n* = 3 per group. (**G** to **I**) *Runx2*, *Alpl*, and *αSMA* mRNA expression in VSMCs. *n* = 3 per group. Data are presented as mean ± SD. *P < 0.05, **P < 0.01, and ***P < 0.001

Osteoblastic differentiation of vascular endothelial cells occupies a crucial role in vascular calcification process (Durham et al. 2018). This process is accompanied by a loss of the smooth muscle cell marker α-smooth muscle actin (αSMA) and an increase in osteochondral markers (Runt-related transcription factor 2 [Runx2], bone morphogenetic proteins [BMP2]), osteopontin, osteocalcin, and alkaline phosphatase [ALP]) (Hortells et al. 2018). Since NaAce inhibits VD-induced vascular calcification in vivo, we then analyzed its effect on osteoblast differentiation in VSMCs. ALP staining showed that OS significantly elevated ALP content in VSMCs, while NaAce treatment significantly inhibited this process (Fig. 5A and B). Besides, Immunofluorescence results showed that the osteogenic markers RUNX2 and BMP2 were increased after OS induction, and the upregulation of these markers was significantly inhibited by NaAce. (Fig. 5C and D, Fig. S2A and B). Through Western blotting experiments, we also detected that NaAce significantly inhibited the protein expression of RUNX2 and ALPL upregulated by OS (Fig. 5E and F, Fig. S2C and D). Similarly, qRT-PCR analysis showed that NaAce suppressed expression of osteogenesis-related genes (*Runx2*, *Alpl*) and upregulated gene expression of *αSMA* in VSMCs (Fig. 5G to I). Taken together, these evidences suggest that NaAce inhibits osteogenic transformation in VSMCs.

### Proteomic analysis of NaAce-treated and non-treated VSMCs

**Fig. 6:**
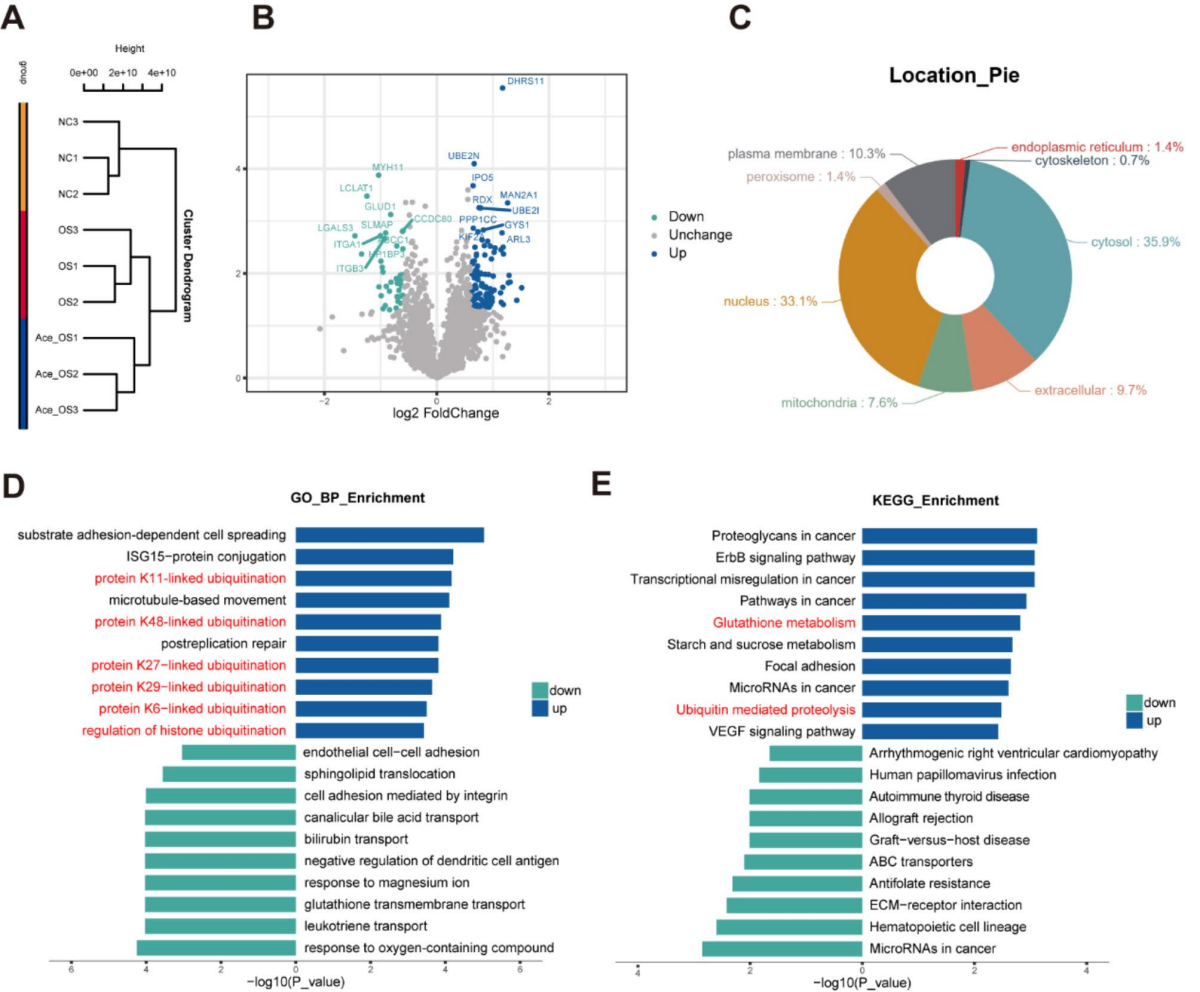
Proteomic analysis of NaAce-treated and non-treated VSMCs. (**A**) Cluster tree for detecting reproducibility of samples in three groups. (**B**) Volcano map showing proteins undergoing up- or down-regulation in OS-induced VSMCs by NaAce intervention. (**C**) Up- or down-regulated proteins resulting from NaAce treatment in terms of subcellular localization GO annotations. (**D**) GO enrichment analysis of differential proteins in the OS + NaAce group relative to the OS group. (**E**) KEGG enrichment analysis of differential proteins in the OS + NaAce group relative to the OS group. *n* = 3 per group. Data are presented as mean ± SD

Label-free proteomics analysis was used to explore the potential mechanisms by which NaAce alleviates osteogenic transformation of VSMCs. First, we performed a repeatability test of the samples by cluster tree analysis, suggesting high similarity within groups and significant differences between groups (Fig. 6A). After NaAce treatment, 107 proteins were upregulated and 38 proteins were downregulated in VSMCs (*P* < 0.05; |fold change| ≥ 1.5) (Fig. 6B). With the help of Gene Ontology (GO) annotation, these proteins were classified according to subcellular localization. As shown in Fig. 6 C, 35.9% of them were from the cytoplasm, 33.1% from the nucleus, and 10.3% were annotated as cytoplasmic membrane proteins. In addition, we analyzed the potential functions of these up- or down-regulated proteins by bioinformatics. GO biological process (BP) enrichment analysis indicated that the proteins upregulated by NaAce were mainly involved in the ubiquitination process of related proteins (Fig. 6D). Kyoto Encyclopedia of Genes and Genomes (KEGG) pathway enrichment analysis showed that proteins up-regulated in the NaAce + OS group relative to the Vehicle + OS group promoted processes such as glutathione metabolism and ubiquitin-mediated protein hydrolysis (Fig. 6E). These findings suggest that the effects of NaAce on the osteogenic transformation of VSMCs may be widespread.

### Glutathione metabolism may be involved in the inhibitory effect of NaAce on osteogenesis

**Fig. 7:**
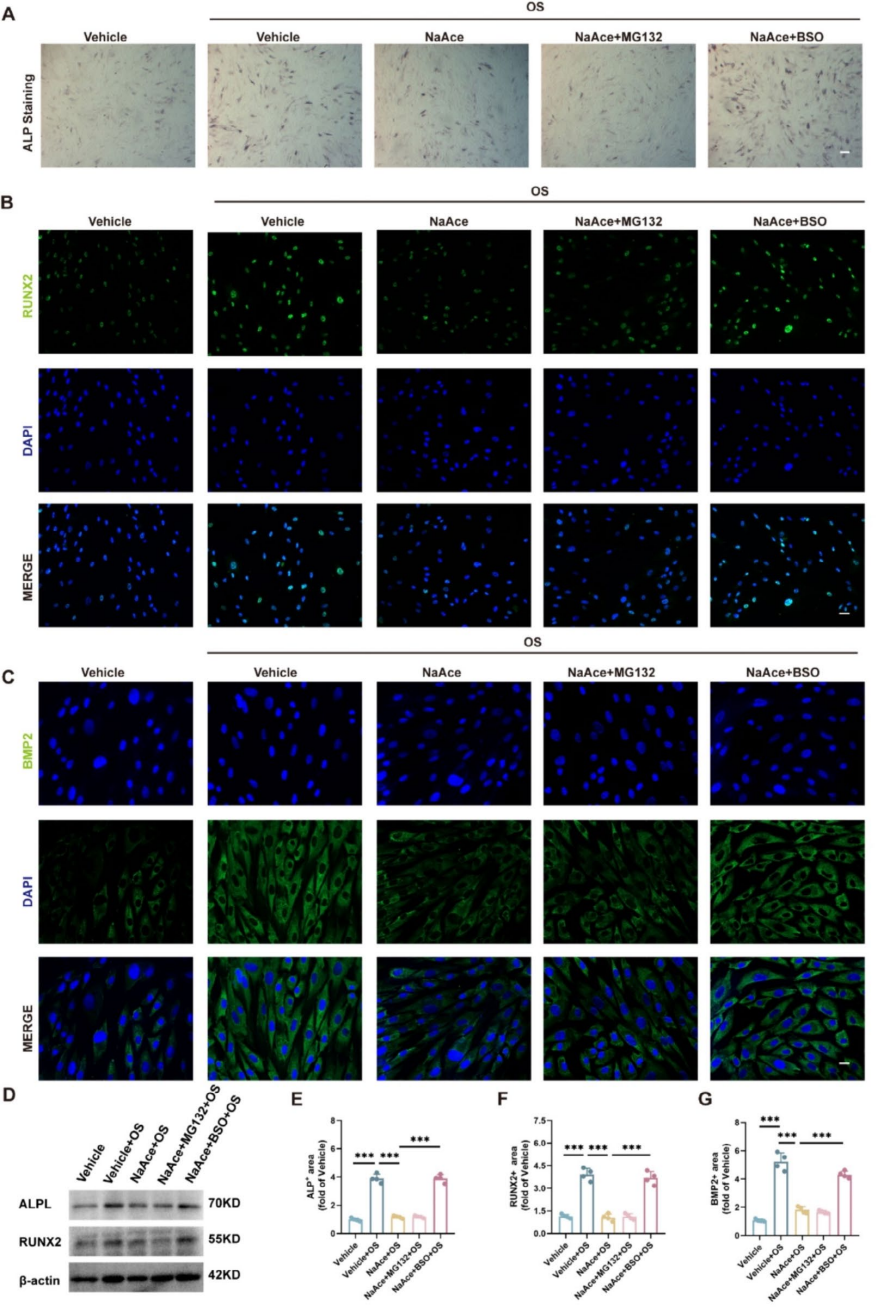
Glutathione metabolism may be involved in the inhibitory effect of NaAce on osteogenesis. (**A**) Alkaline phosphatase (ALP) staining images of VSMCs. Scale bar = 200 μm. (**B**) Immunofluorescence images of Runx2 protein in VSMCs. Scale bar = 20 μm. (**C**) Immunofluorescence images of BMP2 protein in VSMCs. Scale bar = 20 μm. (**D**) Protein expression of Runx2 and ALPL determined using western blotting. *n* = 3 per group. (**E**) Relative quantification of ALP activity. *n* = 4 per group. (**F**) Quantification of relative expression of Runx2 positive area. *n* = 4 per group. (**G**) Quantification of relative expression of BMP2 positive area. *n* = 4 per group. Data are presented as mean ± SD. *P < 0.05, **P < 0.01, and ***P < 0.001

We then used BSO and MG132 to deplete glutathione levels and inhibit ubiquitination-dependent proteasome degradation, respectively. ALP staining results showed that BSO could effectively reverse the inhibition of VSMC osteogenic transformation by NaAce, while MG132 had no effect (Fig. 7A and E). Immunofluorescence results of RUNX2 and BMP2 showed that NaAce significantly inhibited protein expression, while BSO reversed this effect (Fig. 7B, C, F and G). Western blotting results showed that the protein levels of RUNX2 and ALPL were significantly decreased under NaAce intervention, but significantly increased after BSO treatment (Fig. 7D).

## Discussion

**Fig. 8:**
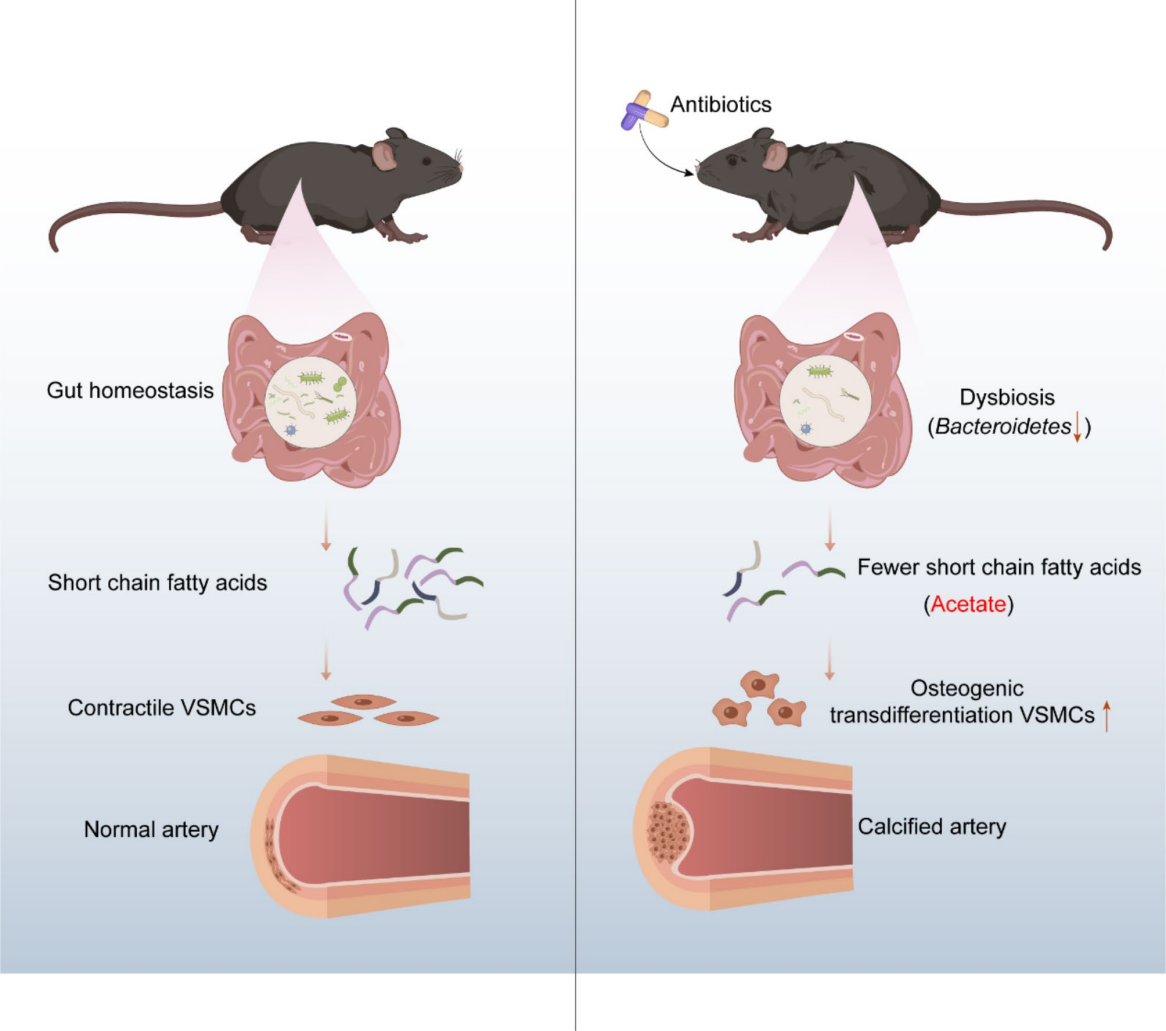
Schematic diagram. Short-chain fatty acid reduction in antibiotic-induced intestinal dysbiosis aggravates vascular calcification

Recently, the crosstalk between GM and cardiovascular disease has been gradually revealed, and an increasing number of studies have moved from association to causality analyses (Kazemian et al. 2020). GM-derived metabolites (SCFAs, TMAO, secondary bile acids, and phenylacetylglutamine) can ameliorate or exacerbate cardiovascular disease through a variety of pathways related phenotypes (including but not limited to atherosclerosis, platelet reactivity, thrombotic potential, hypertension, and vascular inflammation) (Witkowski et al. 2020). Thus, strategies targeting GM are expected to be applied to the management of cardiovascular disease in the foreseeable future. As graphically summarized in Figure 8, we demonstrate for the first time that ABX and vancomycin can exacerbate vascular calcification by disrupting GM. Furthermore, this process was shown to be associated with the inhibition of the production of the SCFAs acetate. These findings may create new ideas for the prevention and treatment of vascular calcification.

Vascular calcification is a cause of high mortality from cardiovascular disease, which is a systemic disease and has a complex and unclear pathogenesis. In order to elucidate its development, several animal models have been used to simulate vascular calcification in previous studies (including naturally occurring animal models, partial nephrectomy, feeding adenine, VD, phosphoric acid, streptozotocin, and genetically modified calcification) (Herrmann et al. 2020). In the present study, we used a VD-induced vascular calcification model to confirm that ABX and vancomycin exacerbate calcification, whereas acetate alleviates calcification. This model is characterized by a short cycle time and a high success rate (Lee et al. 2017). However, whether these results are equally applicable to other scenarios remains unknown and needs to be explored in subsequent studies due to the different mechanisms of vascular calcification in humans and in different animal models of vascular calcification.

The development of antibiotics was undoubtedly one of the greatest medical advances of the 20th century, and in recent years the use of antibiotics has increased worldwide (Browne et al. 2021). Although most courses of antibiotic therapy have no significant adverse effects, antibiotics can lead to significant changes in GM with long-term effects on human health (Dethlefsen and Relman 2011; Haak et al. 2019). First, antibiotic use has been associated with a reduction in GM diversity. The decline in microbial diversity caused by antibiotics has been reported to take at least 1 month to recover (Yassour et al. 2016). In our study, both ABX and single-antibiotic treatments led to a decrease in α-diversity and a significant change in β-diversity in the fecal microbiota of mice. This demonstrates antibiotic treatment disrupts GM composition and structure. Previous studies have shown that age (Murphy et al. 2010), host genetics (Cortes-Ortiz and Amato 2021) and diet can affect the composition of the GM (Murphy et al. 2010; Hildebrandt et al. 2009). Disturbances in composition and, in turn, functionality of the intestinal microbiota can disrupt gut barrier function, a trip switch for metabolic endotoxemia. This low-grade chronic inflammation, brought about by the influx of inflammatory bacterial fragments into circulation through a malfunctioning gut barrier, has considerable knock-on effects for host health. Different mouse models reveal that inflammasome-deficiency-associated changes in the configuration of the gut microbiota are associated with exacerbated hepatic steatosis and inflammation (Henao-Mejia et al. 2012). A change in diet can shift the composition of the gut microbiome rapidly, often within 24 h, in both humans and mice (Murphy et al. 2010; Turnbaugh et al. 2009; Wu et al. 2011). The animal-based diets increased the abundance of bile-tolerant microorganisms, including *Bacteroides*, and decreased levels of *Firmicutes* that metabolize dietary plant polysaccharides (David et al. 2014). There may be multiple factors that interact with the GM disrupted by antibiotics to affect vascular health, which requires more research in the future.

Furthermore, we found that vancomycin intervention significantly reduced the abundance of *Bacteroidetes* in the fecal microbiota and replicated the exacerbation of vascular calcification by ABX. In fact, this is similar to the results of previous studies (Ray et al. 2021; Opstal et al. 2016). Considering that both ABX and vancomycin interventions specifically reduced the abundance of *Bacteroidetes* and aggravated vascular calcification, we hypothesized that the reduced abundance of *Bacteroidetes* may be the key reason for the aggravation of vascular calcification. More interestingly, Villani et al. found that vancomycin can promote the deposition of hydroxyapatite (Hofmann et al. 2021). Eder et al. found that vancomycin affects bone regeneration in vitro (Eder et al. 2016), and the vascular calcification level of osteoporosis patients is higher than that of patients with normal bone density (Lampropoulos et al. 2012; Persy and D’Haese 2009). We speculate that this may be because vancomycin affects calcium formation in bones, thereby increasing free calcium and then ectopically depositing it in blood vessels.

SCFAs are metabolites of indigestible carbohydrates produced by GM fermentation and have been shown to be closely associated with the health of the body (including but not limited to maintenance of intestinal integrity, regulation of glucose homeostasis, modulation of lipid metabolism, modulation of the immune system, and inflammatory response) (Morrison and Preston 2016). Previous studies have shown that *Bacteroidetes* are one of the major contributors to the production of SCFAs in the gut (Aoki et al. 2021; Portincasa et al. 2022). In this study, we found that the concentrations of SCFAs (acetate, propionate, and butyrate) in the serum of mice were significantly reduced after the reduction of the relative abundance of *Bacteroidetes* in the intestine using ABX or vancomycin. Furthermore, we demonstrated that acetate alleviates vascular calcification by inhibiting osteogenic transformation of VSMCs. These findings not only validate the results of previous studies, but also demonstrate that the exacerbation of vascular calcification due to decreased abundance of *Bacteroidetes* is at least partially related to acetate. However, it is noteworthy that a previous study found that 0–10 mM acetate did not affect high Pi-induced calcification of mouse-derived VSMCs in vitro (Zhong et al. 2022). In the present study, we found that 10 mM acetate alleviated OS-induced calcification of human-derived VSMCs. We speculate that differences in cellular origin may be responsible for the different vascular calcification phenotypes.

Dietary fiber influences the composition of the murine GM, helping to maintain the diversity of the community (Sonnenburg et al. 2016) and leading to the production of SCFAs via fermentation (Tanes et al. 2021). Also, a rich fiber diet was shown to improve glucose control, reduce inflammatory response and protect blood vessel walls (Armet et al. 2022; Xiao et al. 2024). Supplementing probiotics such as lactic acid bacteria, bifidobacteria, etc., as well as prebiotics such as inulin and pectin, can support the growth of beneficial intestinal bacteria and promote the balance of GM (Sanders et al. 2019; Żółkiewicz et al. 2020). More recently, aerobic exercise has been shown to impact the gut by increasing microbiome diversity and functional metabolism in both humans and mice (Clarke et al. 2014). Altering the bacterial profiles and influencing the by-products produced from GM through exercise may have the potential to reverse the conditions associated with obesity, metabolic diseases, poor diet, along with neural and behavioral disorders (Monda et al. 2017; Dalton et al. 2019). Therefore, a combination of dietary modification, probiotic supplementation, exercise intervention, and other methods to regulate GM may have the effect of preventing or reversing vascular calcification.

Osteogenic transformation of VSMCs is one of the key factors in vascular calcification formation, which is associated with multiple metabolic processes, and protein modifications (Ouyang et al. 2021, 2022). For example, inhibition of the anti-transporter protein SLC7A11/glutathione/glutathione peroxidase 4 axis drives iron death in VSMCs to promote vascular calcification (Ye et al. 2022), whereas 2-Oxothiazolidine-4-carboxylic acid inhibits vascular calcification by inducing glutathione synthesis (Patel et al. 2021). In vitro and in vivo experiments also showed that ubiquitin proteasomal degradation of histone deacetylase 1 and Runx2 alleviated vascular calcification (Kwon et al. 2016; Huang et al. 2023). Interestingly, using proteomics and bioinformatics, we found that sodium acetate promotes glutathione metabolism and ubiquitin-mediated proteolysis in VSMCs. This evidence suggests that acetate may inhibit osteogenic transformation of VSMCs by affecting these pathways. To verify whether acetate affects the osteogenic transformation of VSMCs through glutathione metabolism and ubiquitination pathways, we used the inhibitor of glutamylcysteine ​​synthetase Buthionine sulfoximine (BSO) to deplete glutathione levels and the proteasome inhibitor MG132 to inhibit the degradation of ubiquitinated proteins (Qiang et al. 2023; Yan et al. 2024). Results showed that glutathione depletion reversed the inhibition of VSMC osteogenic transdifferentiation by acetate, but MG132 had no effect, suggesting that acetate may regulate the osteogenic transformation of VSMCs through glutathione metabolism. Acetate affects the change of ubiquitination level, but the change of ubiquitination does not affect the function of acetate. Studies have shown that acetate alleviates oxidative stress by increasing glutathione (GSH) content and superoxide dismutase (SOD) activity (Liu et al. 2017). Sun et al. found that short-chain fatty acids (SCFA) can promote glutamate-glutamine shuttle to regulate neuroenergetics to alleviate AD (Sun et al. 2023). At the same time, glutathione metabolism is closely linked to vascular calcification. High calcium and phosphate downregulated the expression of SLC7A11 (a cystine-glutamate antiporter) and reduced the content of glutathione (GSH) in VSMCs. Supplementation of GSH reduced VSMC calcification (Ye et al. 2022). These data suggest that glutathione metabolism may play an important role in the anti-calcification effect of acetate.

Finally, there are still some limitations that cannot be ignored. Due to the lack of effective means to specifically eliminate *Bacteroidetes*, we are unable to strongly prove the effect of *Bacteroidetes* deficiency on vascular calcification. Besides, altering the dosage or duration of antibiotics and SCFAs may have different effects on vascular calcification and GM composition. These unresolved issues need to be explored in subsequent work.

## Conclusion

In conclusion, the use of antibiotics may aggravate vascular calcification, so we should be more careful in the clinical use of antibiotics. Our study shows that supplementation with acetate has the potential to alleviate vascular calcification, which may be an important target for future treatment of vascular calcification.

### Electronic supplementary material

Below is the link to the electronic supplementary material.

Supplementary Material 1

Supplementary Material 2

## Data Availability

The data will be available upon reasonable request to the corresponding author.
