# Supplementary material for: Antibiotic-induced gut microbiota disruption promotes vascular calcification by reducing short-chain fatty acid acetate
Source: Mol Med. 2024 Aug 24;30:130. doi: 10.1186/s10020-024-00900-0 (PMC11344439; doi:10.1186/s10020-024-00900-0)
Supplement: Supplementary file 2 — Supplementary Material 2 [file 10020_2024_900_MOESM2_ESM.docx]

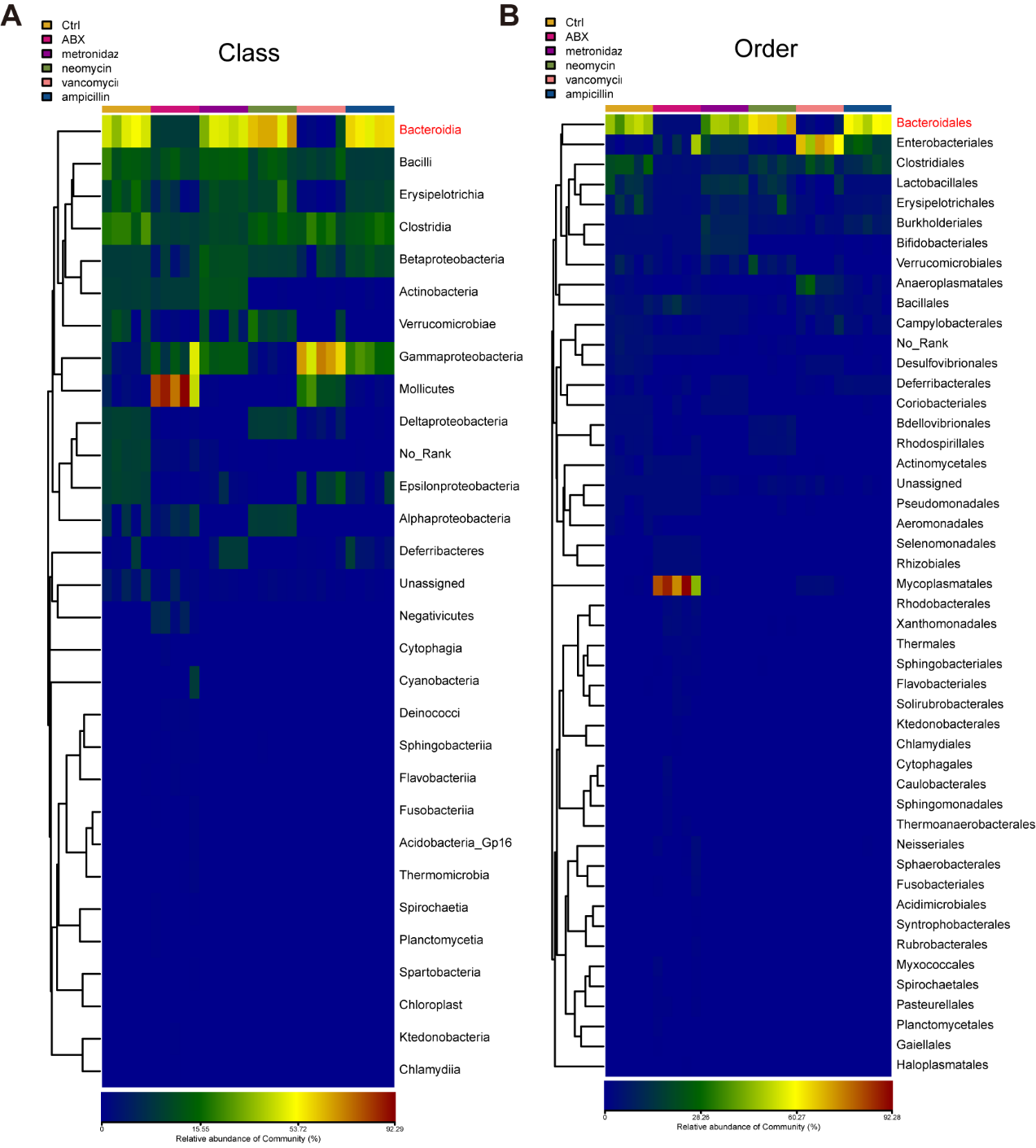
**Fig. S1. Composition of the fecal microbiota of mice detected by 16S rRNA gene sequencing. (A** to **B)** Composition of the mouse fecal microbiota at the class and order and family levels. n = 5 per group.

**
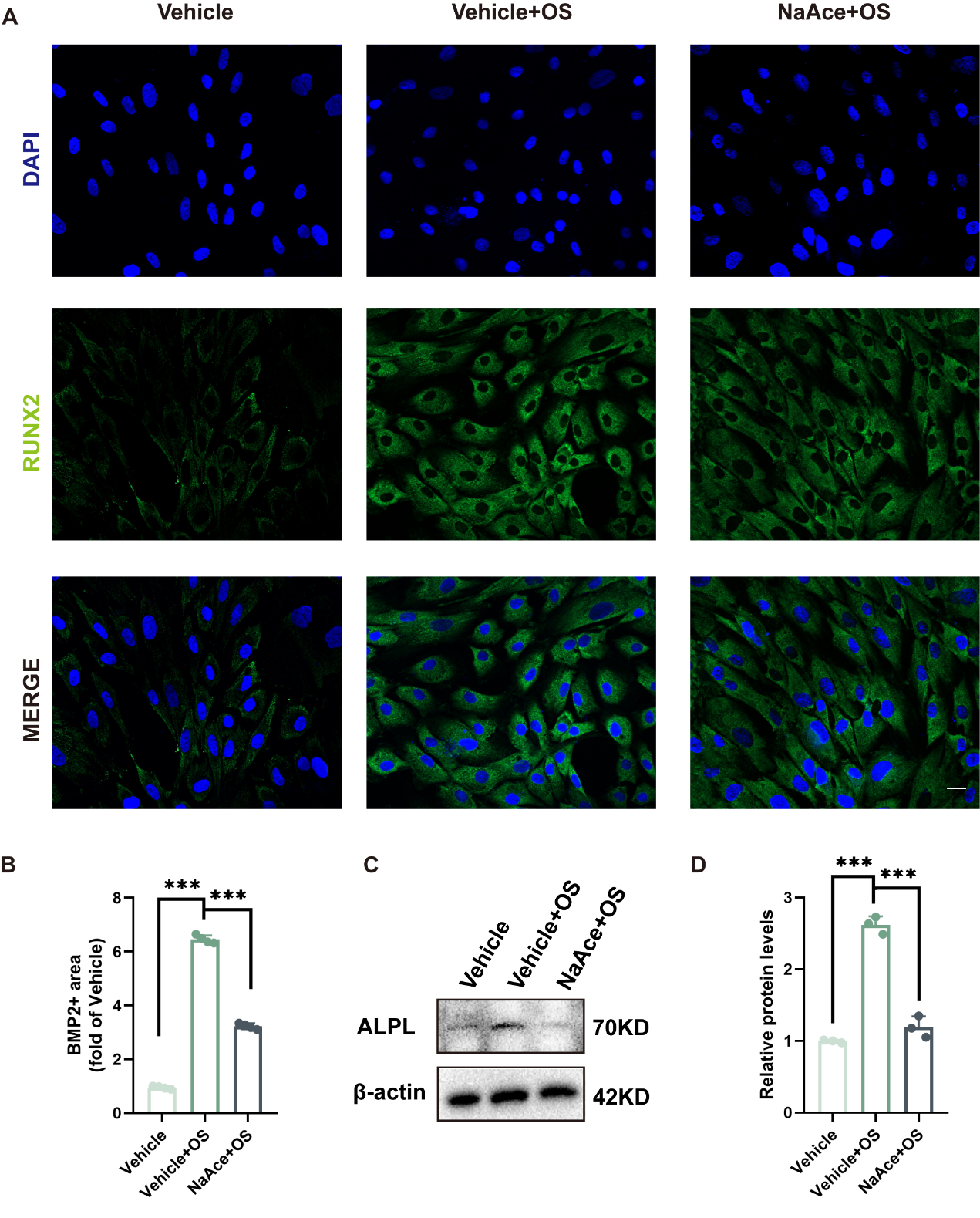
**

**Fig. S2. The expression levels of osteogenic markers BMP2 and ALPL were downregulated under the intervention of NaAce.** (**A**) Immunofluorescence images of BMP2 protein in VSMCs. Scale bar = 20 μm. (**B**) Relative quantification of BMP2 positive area. n = 4 per group. (**C**) Protein expression of ALPL determined using western blotting. n = 3 per group. (**D**) Quantification of relative expression of ALPL. n = 4 per group. Data are presented as mean ± SD. **P* < 0.05, ***P* < 0.01, and ****P* < 0.001.
