# Supplementary material for: Antibiotic-induced gut microbiota disruption promotes vascular calcification by reducing short-chain fatty acid acetate
Source: Mol Med. 2024 Aug 24;30:130. doi: 10.1186/s10020-024-00900-0 (PMC11344439; doi:10.1186/s10020-024-00900-0)
Supplement: Supplementary file 1 — Supplementary Material 1 [file 10020_2024_900_MOESM1_ESM.docx]

Dear editors and reviewers:

Thank you for your letter and for the reviewers’ comments concerning our manuscript entitled “**Antibiotic-induced gut microbiota disruption promotes vascular calcification by reducing short-chain fatty acid acetate**” **(ID: MOME-D-24-00568)**. Those comments are all valuable and very helpful for revising and improving our paper, as well as the important guiding significance to our paper. We have studied comments carefully and have made correction which we hope meet with approval. Revised portion are marked in yellow in the paper. The main corrections in the paper and the responds to the reviewer’s comments are as following:

**Responds to the reviewer’s comments:**

**Reviewer #1:**

1. Reviewer’s comment: “The are a few grammars mistake in the manuscript. Please correct them. As example:

-In the abstract in the results part "ABX exacerbated exacerbated vascular calcification.”

**Response to comment:** Based on your suggestions, We corrected these errors and checked for other similar cases, which are highlighted in the revised manuscript.

2. Reviewer’s comment: “How was the amount of antibiotic cocktail intake controlled in the mice? How could the author ensure a standard antimicrobial concentration intake in the mice? Was the water given at will? This part is not clear, and the results cannot be replicated as described by the author, and it could be subject to bias.”

**Response to comment:** Thank you for your question. As written in the manuscript method, a cocktail of antibiotics (0.5 g/L of vancomycin, 1 g/L of ampicillin, 1g/L of neomycin, 1 g/L of metronidazole) was introduced in drinking water. Line 97-99. The mice were free to drink the water. According to our experience, 5 mice in a cage need to drink 250 mL of water per week. This method is consistent with the experimental protocol reported in the literature (PMID: 36978130) (PMID: 36206317), and this protocol can be replicated.

3. Reviewer’s comment: “Por favor agregue la cita de donde se tomó el modelo de calcificación vascular.”

**Response to comment:** Thank you for your question. We have cited the relevant literature in the revised manuscript. Line 103.

4. Reviewer’s comment: “En la estadística, ¿por qué el análisis intergroup no se hizo con One-way Anova?”

**Response to comment:** Thank you for your suggestion. Actually, the current study used the one-way analysis of variance (ANOVA) test, or Kruskal Wallis test, for the multiple groups according to data type. We're sorry we neglected to describe this in detail. We have described this part in detail in the revised manuscript. Line 196-197.

5. Reviewer’s comment:“In the fecal microbiota transplantation is not clear how was the microbiota depleted material administered orally.”

**Response to comment:** Thank you for your suggestion. In the fecal microbiota transplantation, the feces of mice with or without antibiotic treatment at 4 weeks were collected, the feces of these two groups were centrifuged and resuspended and then administered to recipient mice by oral administration. We have added the description of the relevant content in the revised manuscript. Line 107.

6. Reviewer’s comment:“The abbreviation "VD" is not described in the manuscript, please add its means.”

**Response to comment:** Thank you for your suggestion. We have added the full name of VD in the revised manuscript when it first appeared. Line 85.

7. Reviewer’s comment:“The Alizarin red staining figures are not quantified and is risky made assumptions about qualitative figures. Please add the alizarin red staining quantifications.”

**Response to comment:** Thank you for your suggestion. We have added the quantification analysis of ARS staining in the revised manuscript.

8. Reviewer’s comment:“Please establish how was avoided the inter-individual bias in the stanning figures analysis.”

**Response to comment:** Thank you for your suggestion. In order to avoid individual bias in staining analysis, we usually increase the number of slices of a single sample (3-5 slices), and these slices are uniformly stained and analyzed. The staining results of multiple slices of a single sample are quantitatively analyzed, and the average is taken. Then the average quantitative results of the staining of the individual samples in the group are integrated.

9. Reviewer’s comment:“Agregue las limitaciones del estudio en la discusión.”

**Response to comment:** Thank you for your suggestion. We have added the corresponding discussion in the revised manuscript. Line 399-403.

10. Reviewer’s comment:“Please integrates more the results in the discussion. It would be interesting read what would it be the relation of vancomycin-induced calcium, or if there are reports about metronidazole affecting acetate concentration.”

**Response to comment:** Thank you for your suggestion. We have added the corresponding content in the revised manuscript. Through extensive searches for the relationship between vancomycin and calcium, we found some interesting phenomena. Villani et al. found that vancomycin can promote the deposition of hydroxyapatite (PMID：34680837). Eder et al. found that vancomycin affects bone regeneration in vitro (PMID: 25904413), and the level of vascular calcification in osteoporosis patients is higher than that in patients with normal bone density (PMID: 22890244, PMID: 19733120). We speculate that this may be because vancomycin affects calcium formation in bones, thereby increasing free calcium and then ectopically depositing in blood vessels. Besides, we did not find any articles related to metronidazole and acetate concentration.

**Reviewer #2:**

1. Reviewer’s comment: “The authors should clarify how they validated the vascular calcification model established by intraperitoneally injecting mice with VD (750 U/kg/d) for 4 days, possibly by citing relevant literature or studies that have previously validated similar models. Additionally, while the study observes the effects of treatments over a relatively short duration (4 weeks), it's crucial to address the long-term implications of antibiotic-induced gut microbiota disruption on vascular health. Do these effects persist or worsen over time?”

**Response to comment:** Thank you for your question. We have added references to the vascular calcification model in the revised manuscript. Line103. Whether the effects of antibiotic-induced disruption of the gut microbiota on vascular health persist or worsen over time is an interesting topic worth exploring. In this study, we did not confirm whether these effects persist or worsen over time, which is a limitation of our study. However, there are literature reports that the composition of the gut microbiota of mice gradually changes with the use of antibiotics, At the same time, serum cholesterol and the size of ovarian cancer tumors also gradually increases with the prolonged use of antibiotics (PMID：36423788, PMID: 36206317) . Therefore, we speculate that as the gut microbiota is gradually disrupted during antibiotic use, the impact on vascular health may worsen over time. We will conduct in-depth exploration of this issue in subsequent research.

2. Reviewer’s comment: “The study administers antibiotics and SCFAs at specific doses and durations. However, does altering the dosage or duration of these treatments produce different effects on vascular calcification and gut microbiota composition?”

**Response to comment:** Thank you for your question. We admire your rigorous scientific attitude. Different concentrations may have different effects on vascular calcification and intestinal microbiome composition, but this was not confirmed in our experiments. This is also a shortcoming of our study. We will explore this issue in further research in subsequent studies.

3. Reviewer’s comment: “The study identifies vancomycin as a significant contributor to vascular calcification exacerbation. Are there specific characteristics of vancomycin that make it more detrimental to gut microbiota health compared to other antibiotics used in the study?”

**Response to comment:** Thank you for your question. In our study, we found that compared with other antibiotics, vancomycin has a stronger inhibitory effect on *Bacteroidetes*, which may be an important factor in why vancomycin is more harmful to the health of intestinal microbiota. As for how it affects this bacterium, there is no article reporting yet, which requires in-depth exploration in subsequent studies.

4. Reviewer’s comment: “In the discussion section, the authors should discuss how various factors, including diet, inflammation, and genetic predisposition, interact with antibiotic-induced gut microbiota disruption to impact vascular health.”

**Response to comment:** Thank you for your suggestion. We have added the corresponding discussion in the revised manuscript. Line 327-338.

5. Reviewer’s comment: “In the discussion section, the authors should consider mentioning other interventions targeting gut microbiota modulation that could potentially be equally or more effective in preventing or reversing vascular calcification.”

**Response to comment:** Thank you for your suggestion. We have added the corresponding discussion in the revised manuscript. Line 364-375.

6. Reviewer’s comment: “If SCFAs or other gut microbiota-targeted therapies prove effective in preclinical models, what challenges might arise in translating these findings to clinical practice? How feasible is it to modulate gut microbiota composition and function in human patients with vascular calcification?”

**Response to comment:** Thank you for your suggestion. If SCFAs (short-chain fatty acids) or other gut microbiota-targeted therapies are shown to be effective in preclinical models, translating these findings into clinical practice may face the following challenges. The first is the verification of safety and efficacy. The results in preclinical models need to be further verified in human clinical trials for their safety and efficacy. This includes evaluating the tolerability and potential side effects of different populations. At the same time, the optimal dose, route of administration, and course of treatment need to be determined to ensure that the therapeutic effect is maximized and the risk is minimized. Secondly, the composition and function of the intestinal flora of different individuals vary, which may lead to different responses to SCFAs or specific intestinal microbiota treatments. Therefore, personalized treatment plans need to be developed to target the specific intestinal microbial characteristics of individuals. Finally, cost-effectiveness. The production cost of SCFAs or specific intestinal microbiota treatments may be high, and their cost-effectiveness relative to existing therapies needs to be evaluated.

Studies have shown that SCFAs play an important role in regulating intestinal flora, improving intestinal barrier function and reducing inflammatory response. These findings provide a scientific basis for regulating the composition and function of intestinal flora in patients with vascular calcification. Methods such as fecal microbiota transplantation, probiotic supplementation and dietary intervention have been used to regulate the composition and function of intestinal flora. These methods are relatively mature in technology and have certain feasibility. Regulating intestinal flora may be only one aspect of treating vascular calcification. In practical applications, it may be necessary to combine other treatments (such as drug therapy, surgical treatment, etc.) for comprehensive treatment.

In summary, there are many challenges in translating SCFAs or other intestinal microbiota targeted therapies into clinical practice, but the feasibility of regulating the composition and function of intestinal flora in patients with vascular calcification exists. More research is needed in the future to verify its safety and effectiveness and explore personalized treatment options.

7. Reviewer’s comment: “The authors should incorporate the clinical implications of their findings and acknowledge the limitations of their study for a more comprehensive analysis.”

**Response to comment:** Thank you for your suggestion. We have added the corresponding discussion in the revised manuscript. Line 399-403.

8. Reviewer’s comment: “The authors should carefully review the article to address basic errors, such as the repetition of the word 'exacerbated' in line 32 and the incorrect description of 'cardiovascular' as an organ in line 62.”

**Response to comment:** Thank you for your suggestion. We have made corresponding changes in the revised manuscript based on your suggestions.

**Reviewer #3:**

1. Reviewer’s comment: “alizarin red-staining of tissue sections is too strong, which easily causes artificial signals. It would be better to stain tissues with a lower concentration of dye.”

**Response to comment:** Thank you for your suggestion. We used a standardized protocol for ARS staining, which has been widely used in previous studies (PMID: 37914087; PMID: 34003800), including our group's previous study published in *Nature Communications* (PMID: 35304471).

2. Reviewer’s comment: “The link of observed phenotype with altered glutathione and ubiquitin pathways cannot be established. For example, application of a couple of inhibitors for these pathways in *in vitro* assays would help. ”

**Response to comment:** Thank you for your suggestion. We have supplemented the relevant experiments and described them accordingly in the Results and Discussion. Line 290-297 and 384-398.

3. Reviewer’s comment: “It would be better or convincing to perform more analyses with VSMCs in vitro to observe osteogenic transformation under manipulated conditions, RUNX2 is not enough. In addition, it would be better to isolate vascular endothelial cells and VSMCs from mice to perform proteomics. ”

**Response to comment:** Thank you for your suggestion. We admire your rigorous scientific attitude. We have supplemented the detection of two other osteogenic markers (Alpl\Bmp2), which have been described in the revised manuscript. Line 269-273 and 293-297. In addition, as you said, it would be more ideal to isolate mouse vascular endothelial cells and VSMCs for proteomics research, but considering the time and economic costs, we can only make corresponding improvements in subsequent studies.

4. Reviewer’s comment: “Please provide experimental details for collecting fecal for GM analysis. If not propriate, the GM in collected fecal can be changed artificially. ”

**Response to comment:** Thank you for your suggestion. Before the operation, we read the published literature on GM analysis (PMID: 38266651; PMID: 37054714) and consulted experienced colleagues in our laboratory team who had articles published in *Adv Sci (Weinh)* and *Sci Adv* (PMID: 33977075+PMID: 35417243). Through detailed understanding and learning, we carried out standardized fecal extraction operation. We have added the corresponding details in the revised manuscript. Line154-156.

5. Reviewer’s comment: “Many typos in the manuscript, which have to be corrected. ”

**Response to comment:** Thank you for your suggestion. We have made corresponding changes in the revised manuscript based on your suggestions.

6. Reviewer’s comment: “The quality of images for whole aortas, such as Fig. 1C and H, are low. I would suggest to take multiple images of each aorta with a higher magnification and put images together for one aorta. ”

**Response to comment:** Thank you for your suggestion. Our samples cannot be preserved for such a long time, so we cannot adopt your excellent method in this study. However, we will adopt your suggestions in future work to improve the image quality.

We tried our best to improve the manuscript and made some changes in the manuscript. These changes will not influence the content and framework of the paper. And here we did not list the changes but highlighted in revised paper.

We appreciate for Editors/Reviewers’ warm work earnestly, and hope that the correction will meet with approval. Once again, thank you very much for your comments and suggestions.

Yours sincerely,

Jiang-Hua Liu
